# Effects of family multi-generational relationship on multimorbidity and healthy life expectancy for second generations: insight from the China Health and Retirement Longitudinal Study

**DOI:** 10.1186/s12877-022-03714-z

**Published:** 2023-02-17

**Authors:** Jun-Yan Xi, Si-Rui Zhong, Yu-Xiao Zhou, Xiao Lin, Yuan-Tao Hao

**Affiliations:** 1 Department of Medical Statistics, School of Public Health, Sun Yat-Sen University, 74Th Zhongshan 2Nd Rd, Yuexiu District, Guangzhou, 510080 China; 2 Peking University Center for Public Health and Epidemic Preparedness & Response, Xueyuan Road, Haidian District, 100191 Beijing, China; 3 Sun Yat-Sen Global Health Institute, Sun Yat-Sen University, Guangzhou, 510080 China; 4 Center for Health Information Research, Sun Yat-Sen University, Guangzhou, 510080 China

**Keywords:** Aging, Disease burden, Generational relationship, Healthy life expectancy, Multimorbidity, Non-communicable diseases

## Abstract

**Objective:**

In the context of aging, Chinese families consisting of more than three generations (grandparents, parents, children) are the norm. The second generation (parents) and other family members may establish a downward (contact only with children) or two-way multi-generational relationship (contact with children and grandparents). These multi-generational relationships may have the potential effect on multimorbidity burden and healthy life expectancy in the second generation, but less is known about the direction and intensity of this effect. This study aims to explore this potential effect.

**Methods:**

We obtained longitudinal data from the China Health and Retirement Longitudinal Study from 2011 to 2018, which included 6,768 people. Cox proportional hazards regression was used to assess the association between multi-generational relationships and the number of multimorbidity. The Markov multi-state transition model was used to analyze the relationship between multi-generational relationships and the severity of multimorbidity. The multistate life table was used to calculate healthy life expectancy for different multi-generational relationships.

**Results:**

The risk of multimorbidity in two-way multi-generational relationship was 0.830 (95% *CIs*: 0.715, 0.963) times higher than that in downward multi-generational relationship. For mild multimorbidity burden, downward and two-way multi-generational relationship may prevent aggravation of burden. For severe multimorbidity burden, two-way multi-generational relationship may aggravate the burden. Compared with two-way multi-generational relationship, the second generations with downward multi-generational relationship has a higher healthy life expectancy at all ages.

**Conclusion:**

In Chinese families with more than three generations, the second generations with severe multimorbidity burden may aggravate the condition by providing support to elderly grandparents, and the support provided by offspring to the second generations plays a vital positive role in improving the quality of life and narrowing the gap between healthy life expectancy and life expectancy.

**Supplementary Information:**

The online version contains supplementary material available at 10.1186/s12877-022-03714-z.

## Background

The world population is aging rapidly. The average life expectancy of the population globally reached 72.6 years in 2019, up 8.4 years from 1990, and is expected to reach 77.1 years in 2050 [1]. Rising life expectancy offers a valuable opportunity not only to redefine the possibilities of old age but to give new meaning to extending life [2]. However, the opportunities afforded by increased longevity are largely dependent on health, which is recognized as one key factor [3]. Since the beginning of the twentieth century, chronic diseases, especially multimorbidity, have replaced infectious diseases as the main global health burden, which are closely associated with aging [4]. Multimorbidity burden has become a major issue in the field of public health in the context of population aging, partly due to increased survival rates, improved early disease screening, and lifestyle changes [5]. In a systematic review, Nguyen et al. found that the global prevalence of multimorbidity was about 33.1% with regional variations [6]. Concepció Violan et al. showed that multimorbidity is about 95% common in older age groups [7]. In addition, multimorbidity is associated with many adverse health outcomes in the general elderly population and some specific populations (such as those who suffer from cancer, frailty, diabetes, etc.) [8–10]. These adverse health outcomes include reduced physical function, cognitive function, and quality of life, as well as increased utilization of health resources, and higher rates of mortality [11–15]. In general, multimorbidity is an important health factor that imposes a heavy physical, mental and economic burden on individuals, families, and societies.

As of 2021, the Chinese mainland had 1.43 billion people aged 65 or above, with 13.15 percent of them aged 65 or above. By 2030, the population of the Chinese mainland is expected to reach 1.42 billion, of which 258 million will be over the age of 65, accounting for 18.23 percent of the total population [1]. China is also facing the health challenge of an aging population, with the prevalence of multimorbidity among the elderly in China rising rapidly [16]. Guo et al. found that the prevalence of multimorbidity among the elderly in China is about 49.64%, and the more chronic diseases, the lower the 10-year survival rate [17]. The differences in multimorbidity patterns among the elderly in China may be related to several factors. A higher prevalence of multimorbidity was found in women, higher socioeconomic groups, and higher educational levels, and multimorbidity is also more common among older people in poorer areas than those in wealthier areas [18]. In addition, although associated cultural values and family norms vary from one society to another, family multi-generational relationships are supposed to play a pivotal role in influencing health [19]. The intergenerational family network has become a common family structure in China under the context of population aging, where co-residence or living apart but nearby are regarded as ideal living arrangements [20]. Multi-generational family structures typically include three generations (grandparents, parents, children), or four generations (grandparents, parents, children, grandchildren), in which case the second generations (parents) may play a meaningful role in the intergenerational relationship network [19]. The second generations not only take care of their elderly grandparents but also establish emotional connections and material intercourse with young children and grandchildren. Although previous studies have noticed that intergenerational relationships may have a potential health effect on the second generations in intergenerational family networks, including potential risks or benefits, less is known about the direction and strength of this effect [21–26].

A series of research questions in this study are raised in the context of Chinese families of more than three generations: a) multi-generational relationship was associated with the risk of multimorbidity for the second generations, b) multi-generational relationship was associated with the severity of multimorbidity for the second generations, and c) multi-generational relationship was associated with the healthy life expectancy and life expectancy for the second generations.

## Methods

### Data source and study population

The China Health and Retirement Longitudinal Study (CHARLS) is a longitudinal study of individuals over age 45 in China [27]. The survey includes a rich set of questions regarding economic standing, physical and psychological health, demographics, and social networks of nationally representative aged persons. The CHARLS conducted the baseline survey in 2011 and followed it up in 2013, 2015, and 2018. A detailed description of the CHARLS was published previously [19]. For this study, inclusion criteria were: a) respond to more than two surveys, b) age at baseline ≥ 45 years, c) at least one living parent or parent-in-law, d) at least one living child, and e) contact with children weekly. In the Harmonized CHARLS, a total number of 6,799 out of 25,586 respondents met the inclusion criteria. Among them, 23 respondents were excluded because of abnormal data on follow-up outcomes, and another 8 respondents were dropped because of missing data on any covariate. The final sample size used in the study is 6,768.

### Outcome definition and multimorbidity

The respondent’s history of chronic diseases was based on their answer to the question regarding whether or not a doctor has told the respondent they had a specific medical condition. We defined multimorbidity using two measures. The first measure is two or more concurrent chronic diseases in the same individual, without considering the effect of disease on function [28]. However, simply calculating the number of chronic diseases cannot fully reflect the degree of influence on the quality of life, and the influence of multimorbidity on health resource utilization and medical cost cannot be investigated solely based on the number of diseases [29]. Therefore, the second measure is cumulative indices that incorporate both the number and severity of concurrent diseases. Hu et al. developed the multimorbidity-weighted index, which covers the major chronic diseases with high incidence and severely affecting the quality of life for the middle-aged and elderly in China [30]. The individual multimorbidity burden index was calculated by adding up all the weighted chronic diseases.

### Variable of interest and covariates

The variable of interest was based on the family respondent being asked about contact frequency with both his/her own and their spouse’s mother, father, or children by any means of communication. We defined: a) weekly contact only with children as downward multi-generational relationship; and b) weekly contact with grandparents and children as two-way multi-generational relationship. All models were adjusted for age at interview, gender, living in urban or rural, education, current marital status, participation in social groups, physical activity or exercise, drinking, smoking, and total household per capita consumption.

### Statistical analysis

First, we used the Cox proportional hazards regression, with time since baseline assessment as the start of follow-up, to assess the association between multi-generational relationships and multimorbidity. The time to multimorbidity was derived as the difference between the time of second diagnosis and baseline. Hazard ratios (*HRs*) and corresponding 95% confidence intervals (*CIs*) were calculated, and Schoenfeld's residuals were used to verify the proportional hazard assumption. Secondly, the Markov multi-state transition model was used to estimate the probability of transition between multimorbidity levels. We used the method of exhaustion to list the chronic disease clusters and calculate the multimorbidity burden index for each cluster. The k-means clustering algorithm was used to divide the index into five levels (S1 to S5), with higher levels representing a more severe burden of multimorbidity, and the sixth level (S6) being death. This study assumes that the multimorbidity burden may remain constant or shift to a higher level without reversal because the history of chronic diseases was retrospective. Third, we estimated the probability of transition for multimorbidity burden levels stratified by age as well as variables of interest and calculated the years of healthy life lost due to multimorbidity with the difference in life expectancy and healthy life expectancy using the multistate life tables. All tests used the two-tailed tests, and *P* < 0.05 was considered statistically significant. Detailed modeling information is provided in the Appendix.

## Results

### Characteristics of the study participants

Among the 6768 participants, 2715 (40.1%) had a downward multi-generational relationship, and 4053 (59.88%) had a two-way multi-generational relationship. Statistical differences between covariates were shown in age, gender, marriage status, education, live in urban or rural, drinking, and smoking (Table 1).

**Table 1:** Characteristics of participants by multi-generational relationship [No. (%)]

	Downward multi-generational relationship (*n* = 2715)	Two-way multi-generational relationship (*n* = 4053)	$\chi$ ^*2*^	*P-value*
**Age**				
≤ 65 years	2504 (92.2)	3912 (96.5)	59.900	** < 0.001**
> 65 years	211 (7.8)	141 (3.5)		
**Gender**				
Man	1348 (49.7)	2087 (51.5)	2.136	0.144
Woman	1367 (50.3)	1966 (48.5)		
**Marriage status**			10.164	** < 0.001**
Partnered	2594 (95.5)	3933 (97.0)		
Without partner	121 (4.5)	120 (3.0)		
**Education**				
Less than lower secondary	2320 (85.5)	3315(81.8)	17.275	** < 0.001**
Upper secondary & vocational training	337 (12.4)	650 (16.0)		
Tertiary	58 (2.1)	88 (2.2)		
**Live in urban or rural**				
Rural	1122 (41.3)	1719 (42.4)	0.745	0.388
Urban	1593 (58.7)	2334 (57.6)		
**Drinking**				
No	1748(64.4)	2503 (61.8)	4.689	**0.030**
Yes	967 (35.6)	1550 (38.2)		
**Smoking**				
No	1616 (59.5)	2417 (59.6)	0.005	0.946
Yes	1099 (40.5)	1636 (40.4)		

### Multi-generational relationships and risk of two or more concurrent diseases

**Fig. 1:**
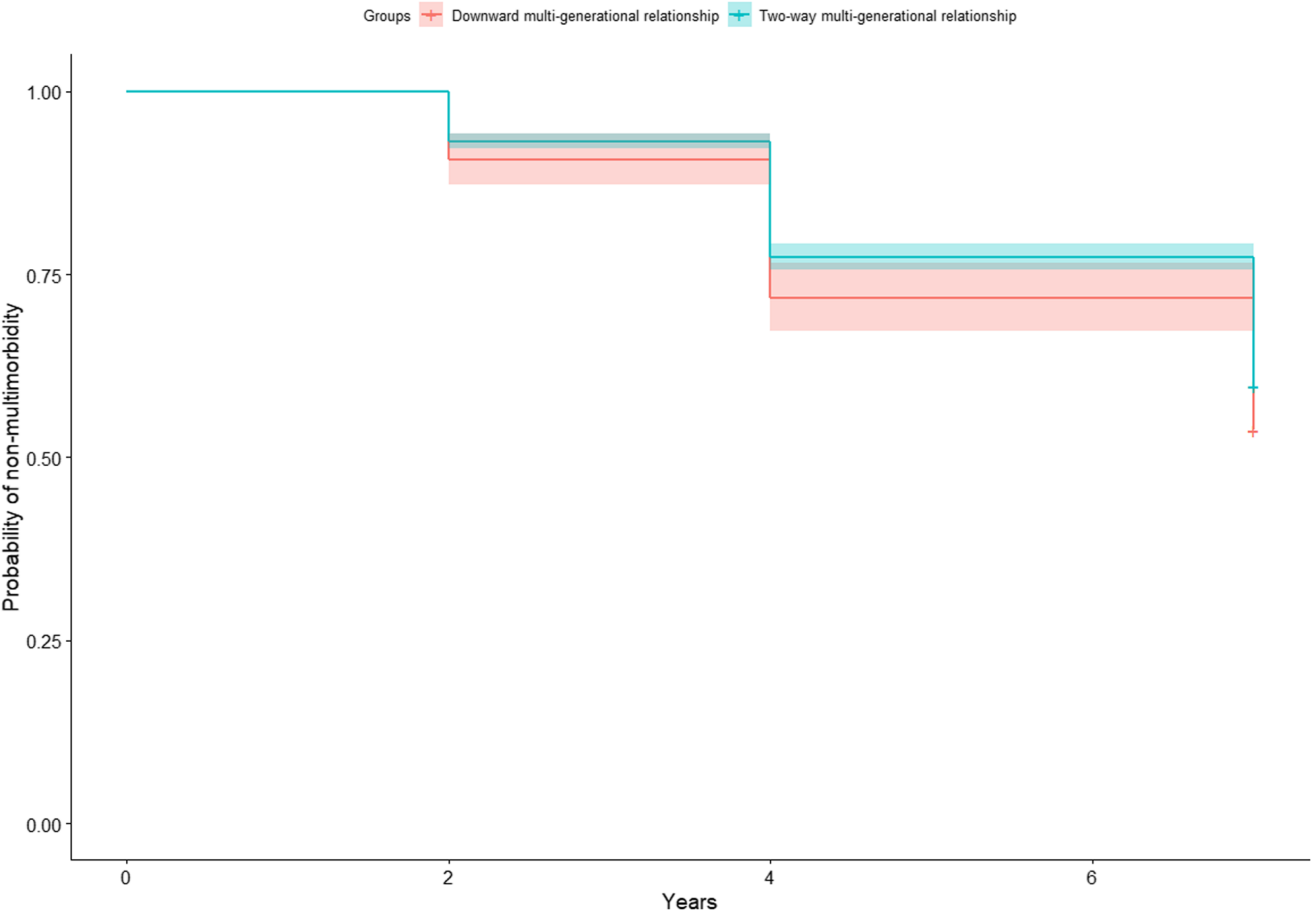
The change of probability without two or more concurrent diseases of people in seven-year-follow-up, group by multi-generational relationship

**Fig. 2:**
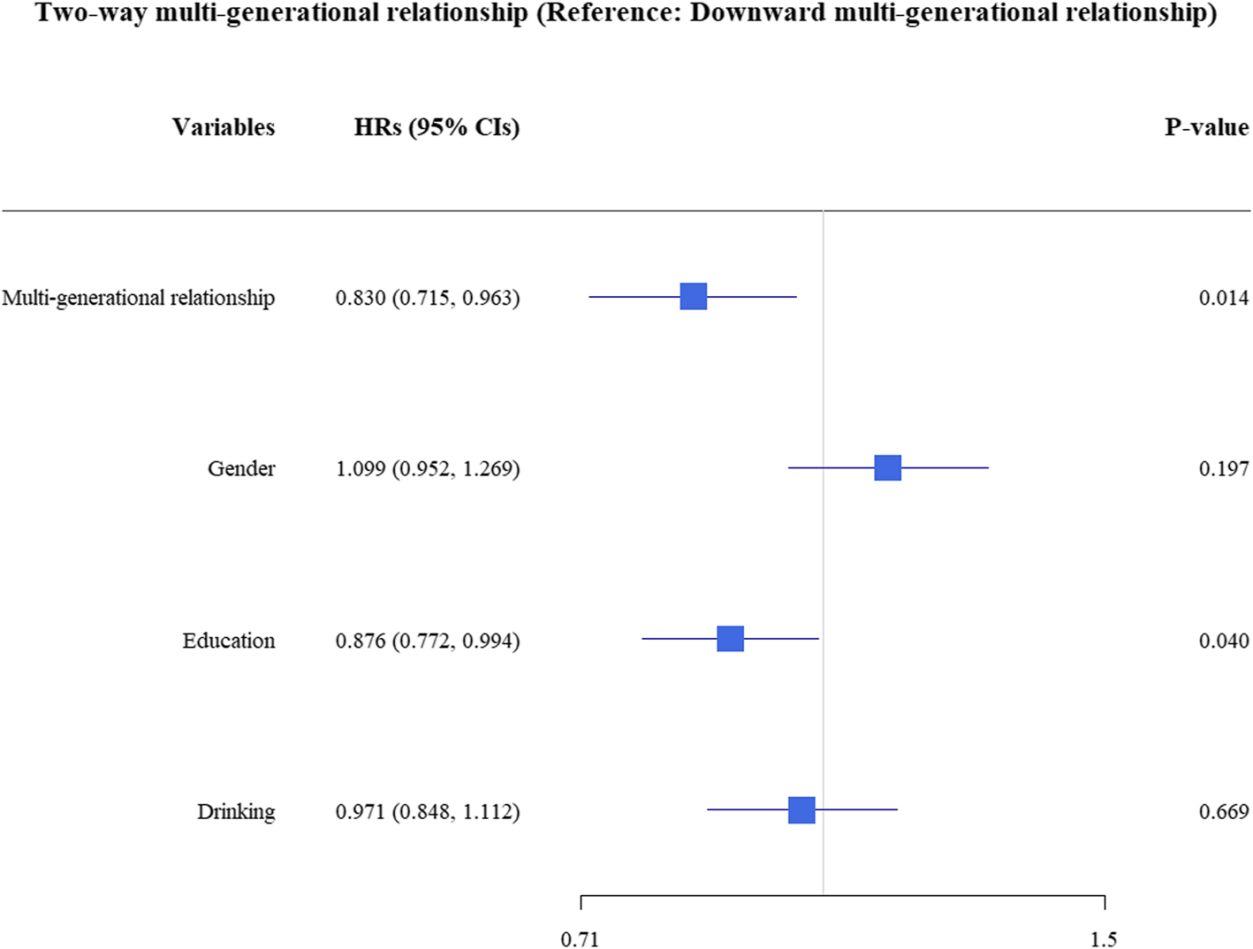
Hazard risks of two or more concurrent diseases, group by multi-generational relationship

In all participants, the probability without two or more concurrent diseases declined continuously during the 7-year follow-up, and the probability of more healthy years without two or more concurrent diseases in two-way multi-generational relationship was greater than that in downward multi-generational relationship (Fig. 1). Overall, the probability without two or more concurrent diseases decreased from 0.907 (95% *CIs*: 0.872, 0.943) to 0.537 (0.496, 0.583) over the 7 years in two-way multi-generational relationship, and from 0.932 (0.921, 0.942) to 0.597 (0.576, 0.618) in downward multi-generational relationship. Compared to the reference group (downward multi-generational relationship), the adjusted *HRs* in two-way multi-generational relationship is 0.830 (*P* = 0.014) (Fig. 2).

### Multi-generational relationships and risk of multimorbidity burden state transition

**Fig. 3:**
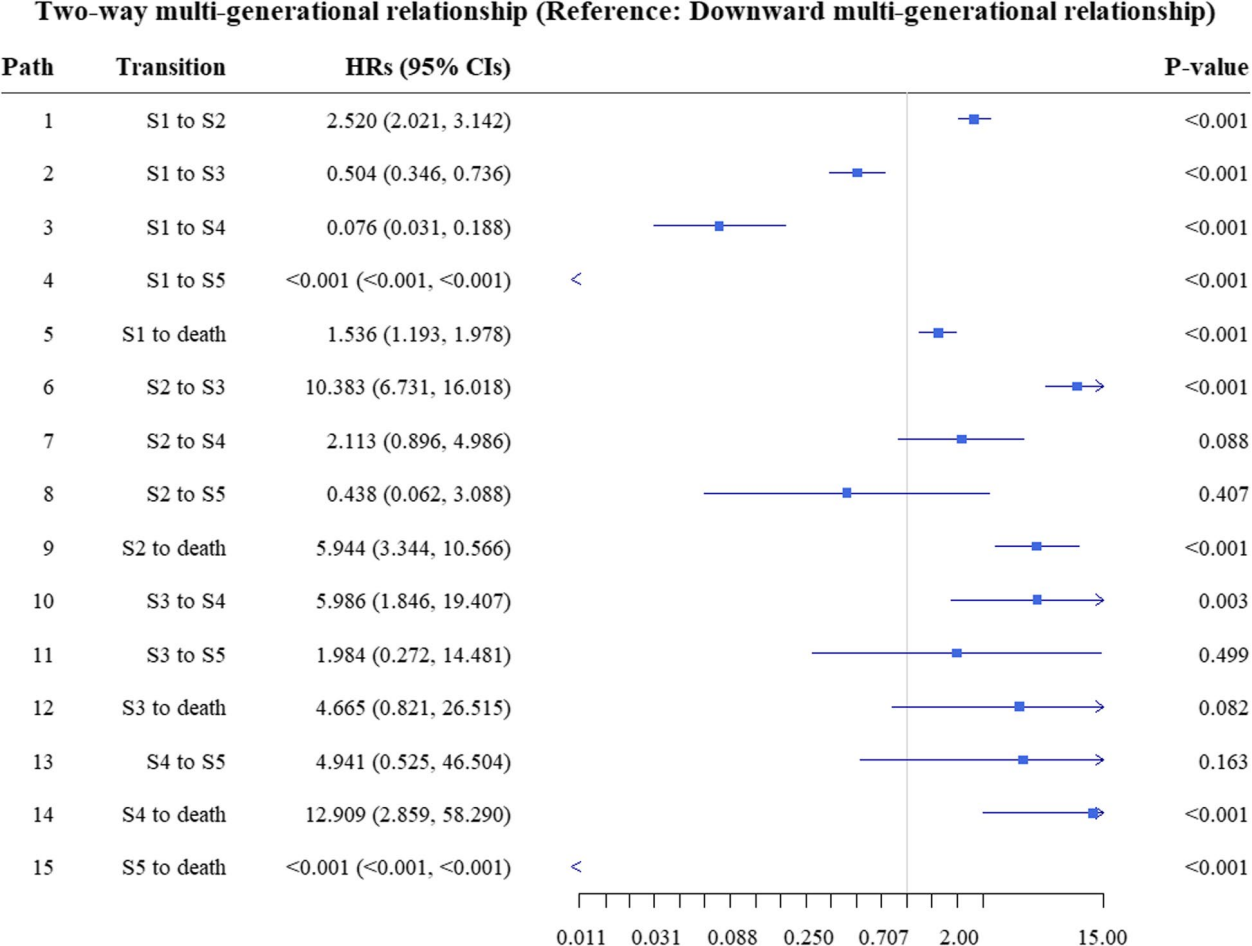
Hazard risks of multimorbidity burden state transition, group by multi-generational relationship

Figure 3 shows the risk of multimorbidity burden state transition based on the Markov multi-state transition model. Compared with the reference group (downward multi-generational relationship), the adjusted *HRs* of path S1 to S3 (*HRs* = 0.504, *P* < 0.001), S1 to S4 (*HRs* = 0.076, *P* < 0.001) and S1 to S5 (*HRs* < 0.001, *P* < 0.001) in two-way multi-generational relationship were less than 1, and those of path S1 to S2 (*HRs* = 2.520, *P* < 0.001), S1 to death (*HRs* = 1.536, *P* < 0.001), S2 to S3 (*HRs* = 10.383, *P* < 0.001), S2 to death (*HRs* = 5.944, *P* < 0.001), S3 to S4 (*HRs* = 5.986, *P* = 0.003), S4 to death (*HRs* = 12.909, *P* < 0.001), and S5 to death (*HRs* < 0.001, *P* < 0.001) were greater than 1.

### Multi-generational relationships and healthy life years

**Fig. 4:**
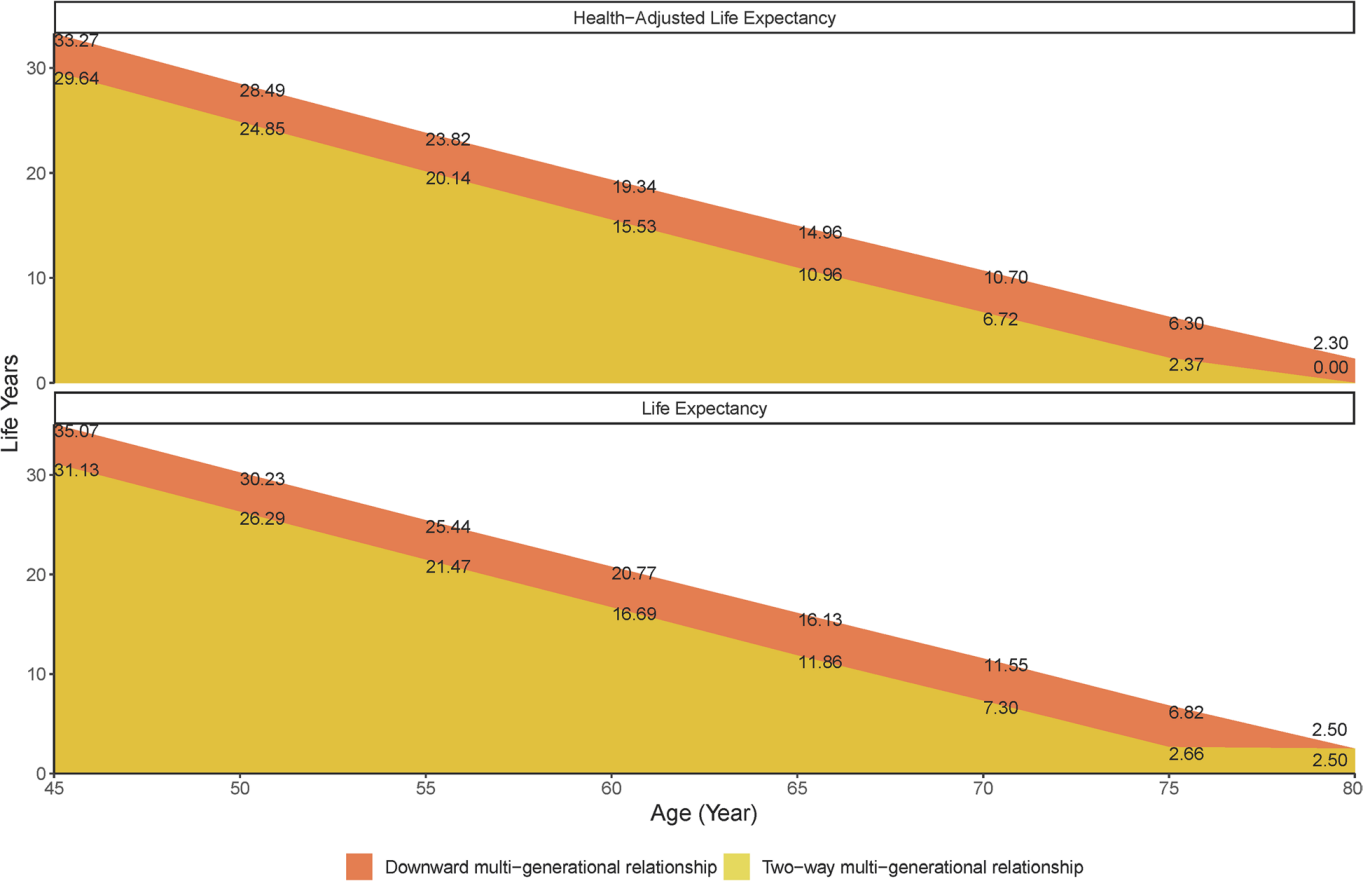
The healthy life expectancy and life expectancy for populations with different multi-generational relationships

Figure 4 shows the healthy life expectancy and life expectancy for populations with different multi-generational relationships. Healthy life expectancy at age 45 was 33.27 years (95% *CIs*: 33.20, 33.34) and 29.64 years (29.58, 29.70) in downward multi-generational relationship and two-way multi-generational relationship, respectively. The life expectancy at age 45 was 35.07 years (35.00, 35.14) and 31.13 years (31.07, 31.20) in downward multi-generational relationship and two-way multi-generational relationship, respectively. Compared with two-way multi-generational relationship, downward multi-generational relationship had a higher healthy life expectancy and life expectancy for all ages.

## Discussion

In China, the most populous country in the world, the health and social problems caused by aging are urgent. Although some studies have revealed a potential relationship between intergenerational networks and health outcomes in family members, the effect on multimorbidity burden and healthy life expectancy for the second generations remains unclear. Our work based on nationally representative survey data addresses this gap and contributes to the literature in important ways. The findings suggest that the second generations who are connected to their children or grandchildren in a weekly manner via multiple communication approaches have the highest healthy life expectancy. However, for the second generations who had two-way connections with both offspring and grandparents, the risk of exacerbation increased if they already suffered from a prior severe multimorbidity.

In general, the health effects of multi-generational relationships on the second generations in Chinese families are characterized differently. One-way relationships with children and grandchildren improve quality of life by reducing the risk of multimorbidity progression, which is confirmed by this study. Evidence from the results of the Survey of Health Ageing and Retirement in Europe suggests that reduced contact with adult children by middle-aged and elderly second generations aged 50 and over affects functional limitations [31]. Pamela L Ramage-Morin et al. found that Métis over the age of 45 who had a strong family network with their children or other adult relatives and could turn to them in times of need were generally more likely to report positive self-perceived health [32]. However, the health benefits of downward multi-generational relationship may be more salient in the Chinese context than in Western countries. These differences in health effects are related to cultural differences, which were partly explained as a relative major of the norms of reciprocity and social harmony in Asian cultures [33]. The patterns characterized by mutual aid and interdependence across generations are prevalent in Chinese families, especially in rural areas [19]. The second generations in such families tend to place a stronger emphasis on family solidarity, harmony, and continuity than their peers in Western cultures. The mental health of older Chinese who had been married and had children benefited most from strong family ties than those from friendships and social communication [34, 35]. Financial and emotional support from adult children is particularly important for the Chinese second generations, which improved their life satisfaction and cognitive function and reduced depressive symptoms [36]. In short, downward multi-generational relationship brings unique health benefits for the second generations through various psychosocial pathways, including staying active in old age and enhancing their self-efficacy and self-esteem.

Another finding was that two-way multi-generational relationship attenuated the positive health effects of downward multi-generational relationship, one possible reason being the consequent burden of informal caregiving. Caring for grandparents who, like themselves, are experiencing age-related health decline can be physically and emotionally demanding for the second generations. Based on the theory of role strain and role enhancement, when the second generations with multimorbidity burden provide informal care for grandparents exceeds their own physical and psychological resources, role strain can arise and aggravate multimorbidity [37]. Chinese second generations in the extended family system often have to take on two contradictory family-centric tasks at the same time, namely the upward responsibility for taking care of the grandparents and the downward responsibility for caring for grandchildren. In this context, second generations play a central role in supporting the diverse and complex multi-generational family. However, they also are forced to be in an intergenerational relationship full of tension. Taking care of elderly grandparents with chronic illness imposes a well-documented burden on second generations, both in health effects and quality of life [38]. A study of 48 low—and middle-income countries found similarly that informal caregivers who provided help to old or weak relatives were more likely to have physical multimorbidity, especially adult caregivers [39]. Riffin et al. found that the burden of family caregivers for community-dwelling older adults in America is determined more by the characteristics of the caregiver (such as sociodemographic and health characteristics of caregivers) and the provision of caregiving tasks (such as activities of daily living related or dementia) than by the characteristics of care recipient [40]. Investing in the prevention of low-quality informal care is key to supporting second generations who suffer from stress, anxiety, or depression, and targeted interventions for mastery and caregiving competence of caregivers to grandparents have potential benefits for second generations [41, 42].

These findings together highlight the importance of family multi-generational relationships in studying the social determinants of health for family members. On the one hand, the government should encourage adult children to live nearby or together with their elderly parents. There are some difficulties in implementing this policy in China. For example, the coverage of medical insurance policies needs to be improved, and the housing policies cannot meet the needs of multi-generation cohabitation. To promote children in multi-generation families to provide support for their parents, the government should support cross-provincial medical insurance, housing supply, real estate tax, floor area ratio management, and other aspects. At the same time, attention should be paid to the people's discussion of this policy, and public opinion should be actively responded to and communicated with. On the other hand, the social pension policies will help relieve the pressure of the traditional family support model. There are many constraints on the development of the pension industry in China. It is urgent to enhance the supply capacity of the pension industry, foster the consumer market of the pension service, perfect the supervision system of the pension industry, and strengthen the construction of talent in the pension industry. The research has important implications for policymakers in China and other countries with similar social contexts.

Some limitations in this study should be acknowledged. First, the CHARLS is ambiguous about the detailed nature and direction of weekly contact with family members, such as greetings or arguments. However, it is not considered a theoretical bias because this connection should be simply regarded as intergenerational cohesion in the view of Chinese filial piety [43]. Frequent contact usually represents a strong emotional bond, the fulfillment of an obligation culturally scripted by filial piety, even if not completely voluntary. Second, it is difficult to obtain a simple upward multi-generational relationship that only provides support to grandparents. Based on traditional Chinese family values, downward multi-generational relationship between offspring and second generations is quite common. Therefore, we use two-way multi-generational relationship as a proxy to explore the health effects of upward multi-generational relationship. This limitation may lead to an underestimation of the health effects in the current study. Future study is needed to design and implement better survey instruments to capture the microscopic networks of relationships between members of the multi-generation family.

In conclusion, the multimorbidity burden and healthy life expectancy of second generations are various among different multi-generational relationship patterns in Chinese families. Although China has made efforts to implement a series of policies to promote healthy aging, such as integrating medical care into ordinary care for the elderly and developing vigorously the sharing aging industry, physical and mental health of family members is still largely determined by the family multi-generational relationship [16, 44, 45]. Any new policy design needs to consider the expectations of heterogeneous subgroups in Chinese families and be tailored to the relatively disadvantaged subgroup.

## Supplementary Information

**Additional file 1.**

## Data Availability

Publicly available datasets were analyzed in this study. The full dataset can be obtained from http://charls.pku.edu.cn/en/.
