# Supplementary material for: Effects of family multi-generational relationship on multimorbidity and healthy life expectancy for second generations: insight from the China Health and Retirement Longitudinal Study
Source: BMC Geriatr. 2023 Feb 17;23:100. doi: 10.1186/s12877-022-03714-z (PMC9938571; doi:10.1186/s12877-022-03714-z)
Supplement: Supplementary file 1 — Additional file 1. [file 12877_2022_3714_MOESM1_ESM.docx]

**Effects of family** **multi-generational relationship** **on multimorbidity and healthy life expectancy for second generations: insight from the China Health and Retirement Longitudinal Study**

**Appendix**

**Contents**

[Cox proportional hazards regression 1](#_Toc7953)

[Multi-state Markov model 4](#_Toc9518)

[Multistate life tables 7](#_Toc18939)

[Multimorbidity-weighted index 9](#_Toc6612)

[Construction of variables of interest 10](#_Toc1144)

# Cox proportional hazards regression

We used a subset of the data to implement survival analysis. Only the responders with no chronic disease or only one chronic disease at baseline were included in the subset. The event of interest is defined as the occurrence of multimorbidity (i.e., the number of chronic diseases ≥ 2). The samples were 4647.

The inverse probability weighted Kaplan-Meier method, log-rank test, and Cox proportional hazard regression were performed.

The expression of Cox regression is:

$$h(t|Z)=h_{0}(t)\exp(\beta_{1}Z_{1}+...+\beta_{k}Z_{k})$$

Where, $Z_{1} \cdots Z_{k}$are a collection of independent variables and $h_{0}(t)$ is the baseline hazard at time *t*, representing the hazard for a person with the value 0 for all the predictor variables.

The premise of applying the Cox proportional hazards regression model is that the effects of the independent variables should meet the proportional hazards assumption. Sample selection and missing data on outcome variables were accounted for by applying the inverse probability weighting method. The individual-level longitudinal weights were used for modelling changes in multimorbidity over the 7-year follow-up period. These weights were constructed by the CHARLS research team and calculated as the product of the household sample selection weight, an inverse probability weighting factor for household non-response, and an inverse probability weighting factor for individual non-response conditional on household participation. These weights were designed to adjust for individual non-response and longitudinal attrition^[1]^. The result of the inverse probability weighted K-M estimation, and log-rank test are shown in Table S1 and Table S2, respectively. Next, we included statistically significant independent variables in the log-rank test in Cox proportional hazards regression model. Table S3 lists the results of the proportional hazard test, in which we found that all input variables meet the assumption (*P* > 0.1). Cox proportional hazards regression model shows that after adjusting for the effects of covariates, the effect of multi-generational relationship is statistical significance, and the hazard ratio of two-way multi-generational relationship to downward multi-generational relationship is 0.830 (Table S4).

Table S1 Inverse probability weighted estimation of non-multimorbidity in different multi-generational relationships

| Multi-generational relationship | Years | Number of non-multimorbidity (million) | Number of multimorbidity (million) | Non-multimorbidity rate (95% *CI*) |
| --- | --- | --- | --- | --- |
| Downward multi-generational relationship | 2 | 56.382 | 5.269 | 0.907 (0.872, 0.943) |
|  | 4 | 51.111 | 10.678 | 0.717 (0.672, 0.765) |
|  | 7 | 40.435 | 10.130 | 0.537 (0.496, 0.583) |
| Two-way multi-generational relationship | 2 | 89.906 | 6.158 | 0.932 (0.921, 0.942) |
|  | 4 | 83.748 | 14.175 | 0.774 (0.757, 0.791) |
|  | 7 | 69.573 | 159.288 | 0.597 (0.576, 0.618) |

Table S2 Log-rank test results of all included variables

| Variables | $\chi^{2}$ | *df* | *P-value* |
| --- | --- | --- | --- |
| Multi-generational relationship | 6.808 | 1 | **0.009** |
| Age | 1.415 | 1 | 0.234 |
| Gender | 5.662 | 1 | **0.017** |
| Marriage status | 0.284 | 1 | 0.594 |
| Education | 4.834 | 2 | **0.028** |
| Live in urban or rural | 0.794 | 1 | 0.373 |
| Drinking | 5.838 | 1 | **0.015** |
| Smoking | 0.014 | 1 | 0.907 |
| Total household per capita consumption | 2.304 | 1 | 0.104 |

Table S3 Results of proportional hazard test

| Variables | $\chi^{2}$ | *df* | *P-value* |
| --- | --- | --- | --- |
| Multi-generational relationship | 0.077 | 1 | 0.781 |
| Gender | 0.001 | 1 | 0.970 |
| Education | 0.613 | 1 | 0.434 |
| Drinking | 2.682 | 1 | 0.102 |

Table S4 Inverse probability weighted results of Cox regression model

| Variables | $\beta$ | Robust *SE* | *HRs* (95% *CIs*) | *z* | *P-value* |
| --- | --- | --- | --- | --- | --- |
| Multi-generational relationship | -0.186 | 0.076 | 0.830 (0.715, 0.963) | -2.45 | **0.014** |
| Gender | 0.095 | 0.073 | 1.099 (0.952, 1.269) | 1.29 | 0.197 |
| Education | -0.132 | 0.065 | 0.876 (0.772, 0.994) | -2.05 | **0.040** |
| Drinking | -0.030 | 0.069 | 0.971 (0.848, 1.112) | -0.43 | 0.669 |

# Multi-state Markov model

The complete data after preprocessing was used in the multi-state Markov model. According to multimorbidity levels, we defined the lowest level to the highest level as S1 to S5. We also include “death” as the sixth level. In this study, we assume that the comorbidity burden may only remain unchanged or shift to a more advanced state. The transfer path matrix and transfer path diagram are shown below. 1-15 represents the transfer paths.


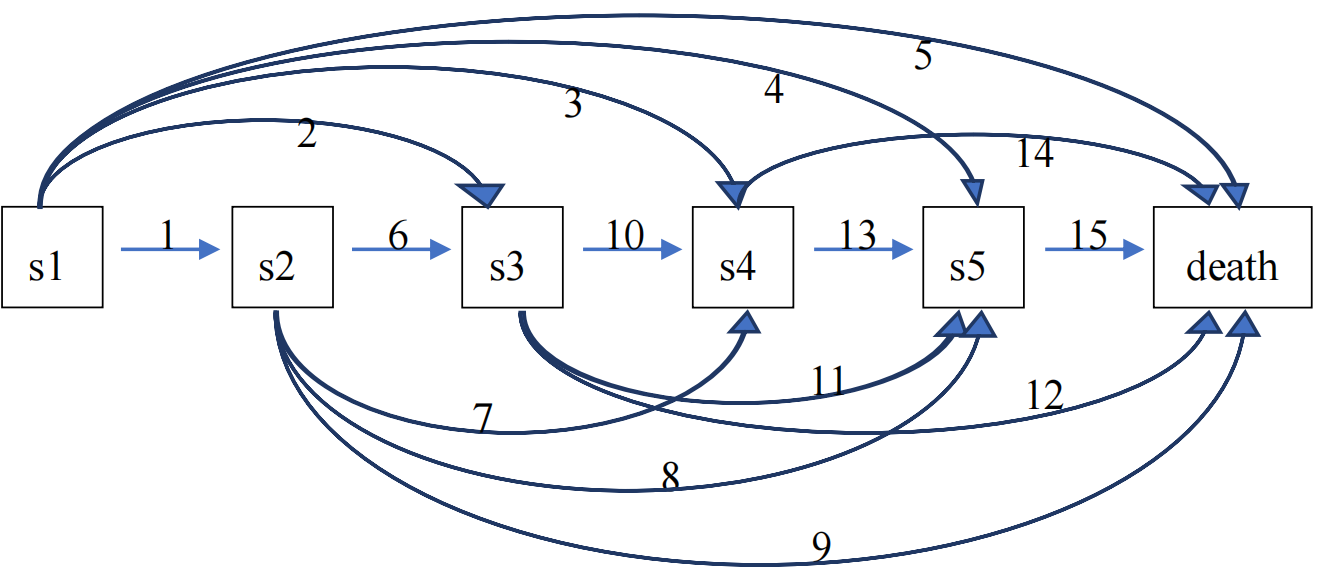


The event of interest for each path is that state transition occurs, in other words, the starting status and the end status of the path existed in the follow-up period of the respondent.

Aalen-Johansen estimator can be used to estimate the transition probability of a Markov process with a finite number of states.

The estimator is given by the expression:

$$T^{lq}(s,t)=\prod_{k=1}^{K} (I^{lq}+\Delta H^{lq}(t_{k}))$$

$$\Delta H^{lq}(t_{k})\text{=}\frac{\Delta N^{lq}(t_{k})}{Y^{l}(t_{k})}$$

where $T^{lq}(s,t)$ denotes the probability that the Markov process starting in state *l* at time *s* will be in state *q* at time *t*. $I^{lq}$ is an identity matrix. $\Delta H^{lq}(t_{k})$ is the number of transitions observed from state *l* to state *q* at the time $t_{k}$ and $Y^{l}(t_{k})$ is the number of entities in state *l* right before time $t_{k}$.

Next, we built the multi-state Markov model. The model expression is:

$$h_{lq}(t)=h_{lq,0}(t)\exp(\beta^{T}Z_{lq})$$

Where, $h_{lq}(t)$ is the hazard function, which is also the transition intensity function of transition from state *l* to state *q* at time *t.* Other equations are similar to the Cox proportional hazards regression model.

The state transition of this data can be described in the following matrix. The results of the estimated transition probability and multi-state Markov model are shown in Table S5 and Table S6.

Table S5 Cumulative transition probability of 15 paths

| State | Years | Cumulative transition probability | | | | | |
| --- | --- | --- | --- | --- | --- | --- | --- |
|  |  | S1 | S2 | S3 | S4 | S5 | death |
| S1 | 0 | 1 | 0 | 0 | 0 | 0 | 0 |
|  | 2 | 0.987 | 0.004 | 0.001 | 0 | 0 | 0.008 |
|  | 4 | 0.957 | 0.197 | 0.002 | 0 | 0 | 0.021 |
|  | 7 | 0.882 | 0.055 | 0.017 | 0.003 | 0 | 0.043 |
| S2 | 0 | - | 1 | 0 | 0 | 0 | 0 |
|  | 2 | - | 0.942 | 0.037 | 0 | 0 | 0 |
|  | 4 | - | 0.845 | 0.091 | 0.007 | 0 | 0 |
|  | 7 | - | 0.592 | 0.187 | 0.053 | 0.013 | 0 |
| S3 | 0 | - | - | 1 | 0 | 0 | 0 |
|  | 2 | - | - | 0.958 | 0.042 | 0 | 0 |
|  | 4 | - | - | 0.870 | 0.072 | 0 | 0.058 |
|  | 7 | - | - | 0.618 | 0.137 | 0.044 | 0.201 |
| S4 | 0 | - | - | - | 1 | 0 | 0 |
|  | 2 | - | - | - | 0.833 | 0.167 | 0 |
|  | 4 | - | - | - | 0.833 | 0.167 | 0 |
|  | 7 | - | - | - | 0.544 | 0.188 | 0.269 |
| S5 | 0 | - | - | - | - | 1 | 0 |
|  | 2 | - | - | - | - | 1 | 0 |
|  | 4 | - | - | - | - | 1 | 0 |
|  | 7 | - | - | - | - | 0.500 | 0.500 |

Table S6 Inverse probability weighted hazard risks of multimorbidity burden state transition, group by multi-generational relationship (Reference: Downward multi-generational relationship)

| Path | Transition | Two-way multi-generational relationship | | |
| --- | --- | --- | --- | --- |
|  |  | *β* | *HRs* (95% *CIs*) | *P-value* |
| 1 | S1 to S2 | 0.924 | 2.520 (2.021, 3.142) | **<0.001** |
| 2 | S1 to S3 | -0.684 | 0.504 (0.346, 0.736) | **<0.001** |
| 3 | S1 to S4 | -2.575 | 0.076 (0.031, 0.188) | **<0.001** |
| 4 | S1 to S5 | -15.947 | <0.001 (<0.001, <0.001) | **<0.001** |
| 5 | S1 to death | 0.429 | 1.536 (1.193, 1.978) | **<0.001** |
| 6 | S2 to S3 | 2.340 | 10.383 (6.731, 16.018) | **<0.001** |
| 7 | S2 to S4 | 0.748 | 2.113 (0.896, 4.986) | 0.088 |
| 8 | S2 to S5 | -0.827 | 0.438 (0.062, 3.088) | 0.407 |
| 9 | S2 to death | 1.782 | 5.944 (3.344, 10.566) | **<0.001** |
| 10 | S3 to S4 | 1.789 | 5.986 (1.846, 19.407) | **0.003** |
| 11 | S3 to S5 | 0.685 | 1.984 (0.272, 14.481) | 0.499 |
| 12 | S3 to death | 1.540 | 4.665 (0.821, 26.515) | 0.082 |
| 13 | S4 to S5 | 1.598 | 4.941 (0.525, 46.504) | 0.163 |
| 14 | S4 to death | 2.558 | 12.909 (2.859, 58.290) | **<0.001** |
| 15 | S5 to death | -15.959 | <0.001 (<0.001, <0.001) | **<0.001** |

# Multistate life tables

The respondents were divided into eight age groups based on their baseline age in 2011, at five-year intervals. The lowest age group was 45-49 years old, and the highest age group was 80 years old and above. The multistate life table was compiled according to the following formula, in which S1 to S5 represented the six levels of multimorbidity burden.

a) Number alive at start of interval:

$$l_{x}=l_{x}^{s1}+l_{x}^{s2}+l_{x}^{s2}+l_{x}^{s3}+l_{x}^{s4}+l_{x}^{s5}$$

b) The number alive at start of interval with different levels of multimorbidity burden, take S1 for example:

$$l_{x}^{s1}=l_{x-n}^{s1}-\left( l_{x-n}^{s1}\times p_{x-n}^{s1 to death} \right)-\left( l_{x-n}^{s1}\times p_{x-n}^{s1 to s2} \right)-\cdots-\left( l_{x-n}^{s1}\times p_{x-n}^{s1 to s5} \right)$$

Where, $p_{x-n}^{s1 to death}$ represents the probability that the number alive at start of interval in S1 may die at the age of $x-n$, and $p_{x-n}^{s1 to s2}$ represents the probability that the number alive at start of interval in S1 may develop into S2 at the age of $x-n$.

c) Number of years lived in interval:

$$L_{x}=\left( l_{x}+l_{x-n} \right)\times n/2$$

d) Number of years lived in interval with different levels of multimorbidity burden, take S1 for example:

$$L_{x}^{s1}=\left( l_{x}^{s1}+l_{x-n}^{s1} \right)\times n/2$$

e) Total number of years lived beyond start of interval:

$$T_{x}=\sum_{x=1}^{w} L_{x}$$

f) Total number of years lived beyond start of interval with different levels of multimorbidity burden, take S1 for example:

$$T_{x}^{s1}=\sum_{x=1}^{w} L_{x}^{s1}$$

g) Average life expectancy at age $x$:

$$e_{x}={T_{x}}/{l_{x}}$$

h) Average healthy life expectancy at age $x$:

$$e_{x}^{s1}={T_{x}^{s1}}/{l_{x}}$$

The state transition probability was estimated by the Markov multi-state transition model stratified by the variable of interest and age groups. The $t$ years' cumulative transition probability ${tp}_{t}$ was converted into a one-year transition probability ${tp}_{1}$ using the following formula:

$${tp}_{1}=1-exp\left( \frac{ln\left( 1-{tp}_{t} \right)}{t} \right)$$

# Multimorbidity-weighted index

The Chinese multimorbidity-weighted index (CMWI) covers the major chronic diseases with high incidence and severely affecting the quality of life for the middle-aged and elderly in China (Table S7)^[2]^.

Table S7 The Chinese multimorbidity-weighted index (CMWI)

| Chronic disease | Index |
| --- | --- |
| Stroke | -5.1 |
| Memory-related disease (e.g. dementia, brain atrophy, Parkinson’s disease) | -4.3 |
| Cancer or malignant tumour (excluding minor skin cancers) | -3.4 |
| Asthma | -2.4 |
| Arthritis or rheumatism | -2.2 |
| Emotional, nervous, or psychiatric problems | -2.1 |
| Heart disease (e.g. coronary heart disease, angina, congestive heart failure) | -1.7 |
| Chronic lung diseases (e.g. chronic bronchitis, emphysema, excluding tumours or cancer) | -1.6 |
| Hypertension | -1.3 |
| Kidney disease (except for tumour or cancer) | -1.1 |
| Diabetes or high blood sugar | -1.0 |
| Stomach or other digestive diseases (except for tumour or cancer) | -0.7 |
| Dyslipidaemia (e.g. elevation of total cholesterol) | -0.2 |
| Liver disease (except fatty liver, tumours, and cancer) | -0.2 |

**Calculation of the CMWI**

a) Assess for the presence of physician-diagnosed chronic diseases.

b) Look up the multimorbidity-weighted index weightings (the Index in Table S7) for each chronic disease.

c) Add up all the weighted chronic diseases to create the CMWI.

Example:

a) Participant A has stroke, memory-related disease, and cancer identified by the doctor.

b) Using Table S7, the CMWI disease weightings corresponding to stroke, memory-related disease, and cancer are 5.1, 4.3, and 3.4, respectively.

c) Person A has a CMWI = 5.1 + 4.3 + 3.4.

# Construction of variables of interest

In CHARLS, the family respondent is asked about contact frequency with both his or her mother and father and their spouse’s mother and father, a variable named HwPCNT in the dataset. HwPCNT indicates whether the family respondent and spouse have any weekly in-person contact with parents/parents-in-law. HwPCNT is assigned 0 if the family respondent reports no weekly in-person contact with parents/parents-in-law. HwPCNT is assigned 1 if the family respondent reports any in-person contact with parents/parents-in-law at least once a week. HwPCNT is also assigned 1 if any parent/parent-in-law co-resides with the family respondent and spouse. Similarly, the family respondent is asked about contact frequency with both his or her children and their spouse’s children, a variable named HwKCNT in the dataset. HwKCNT indicates whether the family respondent and spouse had any weekly contact with any of their children in person or by phone, text message, mail, or e-mail. HwKCNT is assigned 0 if the family respondent reports no weekly contact with their children in person or by phone, text message, mail, or e-mail. HwKCNT is assigned 1 if the family respondent reports any contact with their children in person or by phone, text message, mail, or email at least once a week. HwKCNT is also assigned 1 if any of their children co-reside with the family respondent and spouse^[1]^.

**References**

[1] National School Of Development, Peking University. The China Health and Retirement Longitudinal Study (CHARLS). Available: http://charls.pku.edu.cn/en/. accessed June 30, 2022

[2] Hu WH, Liu YY, Yang CH, et al. Developing and validating a Chinese multimorbidity-weighted index for middle-aged and older community-dwelling individuals. *Age Ageing*. 2022;51(2):afab274. doi:10.1093/ageing/afab274
